# Mean sojourn time, overdiagnosis, and reduction in advanced stage prostate cancer due to screening with PSA: implications of sojourn time on screening

**DOI:** 10.1038/sj.bjc.6604973

**Published:** 2009-03-17

**Authors:** N Pashayan, S W Duffy, P Pharoah, D Greenberg, J Donovan, R M Martin, F Hamdy, D E Neal

**Affiliations:** 1 Department of Public Health and Primary Care, Institute of Public Health, University Forvie Site, Robinson Way, Cambridge CB2 OSR, UK; 2 Cancer Research UK Centre for Epidemiology, Mathematics and Statistics, Wolfson Institute of Preventive Medicine, Charterhouse Square, London EC1M 6BQ, UK; 3 Department of Oncology, University of Cambridge, Strangeways Research Laboratory, Worts Causeway, Cambridge CB1 8RN, UK; 4 Eastern Cancer Registration and Information Centre (ECRIC) Unit C, Magog Court, Shelford, Bottom Hinton Way, Cambridge CB22 3AD, UK; 5 Department of Social Medicine, University of Bristol, Canynge Hall, Whiteladies Road, Bristol BS8 2PR, UK; 6 Nuffield Department of Surgery, University of Oxford, Oxford John Radcliffe Hospital Hospital, Headington, Oxford OX3 9DU, UK; 7 Department of Oncology, University of Cambridge, Addenbrooke's Hospital, Cambridge CB2 2QQ, UK

**Keywords:** prostate cancer, screening, mean sojourn time, overdiagnosis, advanced stage

## Abstract

This study aimed to assess the mean sojourn time (MST) of prostate cancer, to estimate the probability of overdiagnosis, and to predict the potential reduction in advanced stage disease due to screening with PSA. The MST of prostate cancer was derived from detection rates at PSA prevalence testing in 43 842 men, aged 50–69 years, as part of the ProtecT study, from the incidence of non-screen-detected cases obtained from the English population-based cancer registry database, and from PSA sensitivity obtained from the medical literature. The relative reduction in advanced stage disease was derived from the expected and observed incidences of advanced stage prostate cancer. The age-specific MST for men aged 50–59 and 60–69 years were 11.3 and 12.6 years, respectively. Overdiagnosis estimates increased with age; 10–31% of the PSA-detected cases were estimated to be overdiagnosed. An interscreening interval of 2 years was predicted to result in 37 and 63% reduction in advanced stage disease in men 65–69 and 50–54 years, respectively. If the overdiagnosed cases were excluded, the estimated reductions were 9 and 54%, respectively. Thus, the benefit of screening in reducing advanced stage disease is limited by overdiagnosis, which is greater in older men.

Prostate cancer is the most frequently diagnosed cancer in men and the second leading cause of cancer death in men in the industrialised world (Zhu *et al*, 2006). The value of screening using prostate-specific antigen (PSA) testing is still controversial (Ilic *et al*, 2006), nevertheless there is considerable asymptomatic PSA testing in developed countries. To quantify the likely benefits and harms of various PSA testing regimens is relevant to an understanding of the natural history of the disease. A crucial parameter in early detection is the sojourn time, the period in which the tumour is asymptomatic but detectable by screening. This indicates the upper limit of time by which diagnosis is advanced by screening (lead time). Accurate estimation of sojourn time facilitates inference on the optimum interval between screens, the likely effectiveness of screening, and the extent of overdiagnosis. Conceptually, overdiagnosis is the diagnosis due to screening, which would not have led to a clinical diagnosis during the lifetime of the host had screening not taken place (Paci *et al*, 2004).

Mean lead times and sojourn times due to PSA screening have been estimated in retrospective studies that used stored blood samples obtained from individuals who were later clinically diagnosed with prostate cancer (Stenman *et al*, 1994; Auvinen *et al*, 2002) and in simulation studies (Draisma *et al*, 2003; Telesca *et al*, 2008). However, there are no analytic estimates obtained directly from screening data, and there are no estimates from the United Kingdom. The only published UK study on overdiagnosis, reported tentative overdiagnosis estimates attributable to increased diagnostic PSA testing rather than to screening, using estimates of lead time from the literature (Pashayan *et al*, 2006).

In this study we use empirical data to estimate the age-specific mean sojourn time (MST), the subsequent likelihood of overdiagnosis, and the predicted potential relative reduction in advanced stage disease following screening at different intervals. These estimates are derived from prevalence screen-detected cases and incidence of clinical cases using biostatistical and epidemiological methodology, without formal mathematical modelling.

## Materials and methods

Data on prevalent cases were obtained from the Prostate Testing for Cancer and Treatment (ProtecT) study, an ongoing national study of community-based PSA testing and randomised trial of subsequent prostate cancer treatment (Donovan *et al*, 2003). Prostate-specific antigen testing in the context of the Protect study is akin to prevalence screening. In this paper, we refer to ProtecT-detected cases as PSA-detected cases.

In the ProtecT study, approximately 200 000 men between the ages of 50 and 69 years, ascertained through randomly selected general practices in nine regions in the United Kingdom, were invited for enrolment. Men with concomitant or past malignancies or other major comorbidities that precluded enrolment in the treatment trial were excluded. Consenting eligible men were offered a PSA test. A PSA level of 3.0 ng ml^−1^ was used as the threshold for further investigation. All men with PSA ⩾3.0 ng ml^−1^ were offered transrectal, ultrasound-guided biopsy using a 10-core lateral biopsy template. Pathologic evaluation was carried out by specialist uropathologists in each centre. All laboratories have participated in the UK National External Quality Assessment Service programme for PSA testing (Donovan *et al*, 2003).

Data on clinically detected prostate cancer cases were obtained from the Office for National Statistics. These data included number of registered cases in England by year of registration and 5-year age group between 2002 and 2005. Mid-year population estimates for England for each year from 2002 to 2005 by 5-year age groups were obtained from the Office for National Statistics. Age-specific incidence of clinical prostate cancer per 10^5^ person years was calculated. Individual-based data with information on clinical stage and histological grade were obtained from the Eastern Cancer Registry and Information Centre (ECRIC) – population-based cancer registry in the East of England.

Prostate cancer was classified as localised disease with tumour–node–metastasis (TNM; Sobin and Wittekind, 1997) stage T2 and below; and regional-distant (advanced stage) disease with TNM stage T3 and above.

### Statistical analysis

The mathematical details and the relevant formulae are given below. Our broad strategy, however, was as follows:

1. Stratified by age, estimate the MST of prostate cancer, taking account of sensitivity of testing, which we derived from the literature.
2. By combination of (1) with national statistics on death rates by age, estimate the proportion of overdiagnosed tumours by age and testing frequency, that is, those tumours, which would not have become symptomatic before the host died of other causes.
3. From the sojourn time estimates, calculate the expected proportions of screen-detected and interval cancers by age and PSA testing frequency.
4. From the stage distribution of PSA-detected tumours in ProtecT and those in the general population, combined with the results of (3) above, estimate the likely reduction in advanced stage disease by age and testing frequency.
5. Adjust the estimates in (4) by subtracting overdiagnosed cases from the early disease cases (although we estimate overdiagnosis from overall incidence, to obtain conservative estimates of the benefit of PSA testing, we assume that all overdiagnosed cases are localised stage).

### The sensitivity of PSA test

The sensitivity of the PSA test was derived from the literature (Bretton, 1994; Imai *et al*, 1994, 1995; Jubelirer *et al*, 1994; Stenman *et al*, 1994; el-Galley *et al*, 1995; Higashihara *et al*, 1996; Brett, 1998; Gustafsson *et al*, 1998; Hoffman *et al*, 2002; Mistry and Cable, 2003). Age-specific sensitivity values were estimated using a linear regression analysis of reported sensitivity on age at testing and PSA cut-off used, weighted by the number of individuals tested in each study and age group. A clustered regression analysis was used, allowing observations from the same study to be correlated, but assuming independence of observations from different studies.

### Mean sojourn time

The mean sojourn time, the average time spent in the preclinical screen-detectable phase, is assumed to have exponential distribution (Day *et al*, 1989). The instantaneous transition rate from preclinical to clinical disease is *λ*. The inversion of *λ* is the MST. If *S* is the sensitivity of the screening test, *P* is the prevalence of preclinical disease, and *I* is the annual incidence of preclinical disease, then from Paci and Duffy (1991)

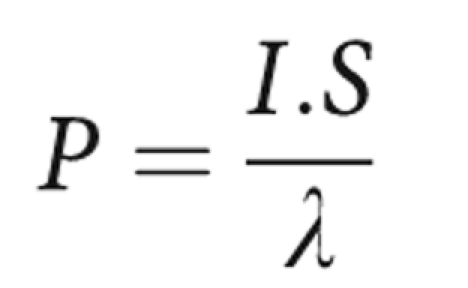


If the MST is *n* years, one would expect to anticipate approximately *n* years of disease incidence with a single screen of a population, assuming good sensitivity (Day *et al*, 1989). As the incidence of prostate cancer changes with age, for each age at screen, MST was calculated as the number of years where the cumulative incidence catches up with the preclinical incidence, such that as *I* changes with age, let (1/*λ*)=*n*, then we find *n* such that 
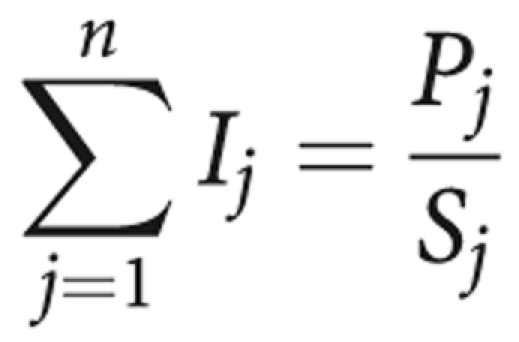
where *I*_*j*_=incidence in year of life *j* and *j*=1 corresponds to age at screen.

The 95% confidence intervals (CI) on the MST were estimated from the 95% CI on *P* assuming a Poisson distribution of prevalence and assuming that the sensitivity did not contribute mutually to the variation.

### Probability of overdiagnosis

The probability of overdiagnosis, probability that a PSA-detected case would have taken longer than the remaining lifetime to progress to clinical cancer, can be estimated as 
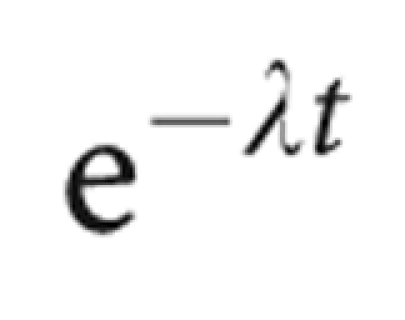
 where *t* is the expected remaining lifetime (Paci *et al*, 2004). Using expected remaining lifetimes for the UK male population in 2003–05, and estimates of age-specific sojourn time, age-specific probabilities of overdiagnosis were calculated.

The 95% CI on the probability of overdiagnosis were estimated from the 95% CI values of *λ*.

### Relative reduction in advanced stage disease by PSA screening

Here we define clinical disease as the disease detected in the absence of screening and preclinical disease as that detected by screening. Using the approach of Launoy *et al* (1998), the probability of diagnosing prostate cancer at screening in a population subjected to screening (as opposed to the disease arising clinically as an interval cancer) was derived. This estimate is often referred to as the programme sensitivity (ps) and depends on the screening sensitivity, the sojourn time, and the interval between screens such that 
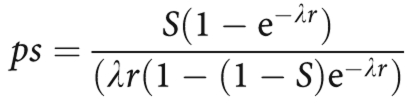
 where *r*=interval between screens

The 95% CI on the estimated ps was calculated using the Delta method.

Knowing the proportions of preclinical and clinical localised stage prostate cancer from the ProtecT and ECRIC databases, respectively, and the probability of diagnosing prostate cancer at screening, it is possible to estimate the expected proportion of localised disease *E*(*p*_*l*_) following screening. *E*(*p*_l_) is the sum of the proportion of localised disease detected by screening and the proportion of localised disease among interval cases 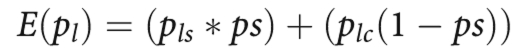 where *p*_ls_ proportion of localised disease detected by screening; and *p*_lc_ proportion of localised disease detected clinically. We estimated *p*_lc_ from the ECRIC data. The expected proportion of advanced disease is *E*(*p*_a_)=1−*E*(*p*_l_). The relative reduction in advanced disease following screening is 
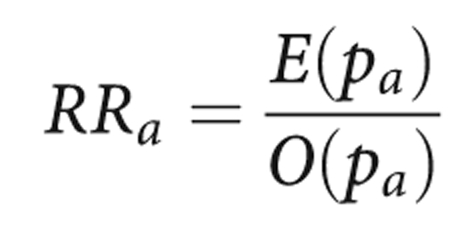
 where *O*(*p*_a_) is the observed proportion of advanced disease detected clinically, also estimated from the ECRIC data. Assuming that overdiagnosis occurs only in the prevalence screening and only applies to localised disease, the relative reduction in advanced disease following screening was estimated after excluding the proportion of overdiagnosed cases.

The 95% CIs on the expected RRs of advanced stage disease were calculated using repeated application of the Delta method to the estimated components, assuming that the proportions with advanced stage disease were binomially distributed. To render tractable the estimation of CIs, we had to constrain at least one estimate as a fixed value and so with PSA test sensitivity as the published estimates were based on large numbers and the standard error would therefore have been very small. Further details of the interval estimation are available from the authors (SD, NP).

## Results

The average uptake of PSA testing within ProtecT was 48% in response to single invitation; 11% of men had an abnormal PSA result, of whom approximately 12% refused biopsy. Between 1 January 2002 and 31 December 2005, 43 842 men were tested. Of those, 1544 (3.5%) were identified with prostate cancer. Table 1 shows the number of persons tested and cases diagnosed by age and stage. The mean age (s.d.) of diagnosis was 62.0 (4.9) years. Of the PSA-detected cancers, 87% were localised. In England, in the same period, 42 850 men, aged 50–69 years, were registered with clinically detected prostate cancer and 27% of the cases presented with advanced stage disease (ECRIC data; Table 1).

Appendix A summarises the studies used to derive the sensitivity of PSA test at cut-off of 3.0 ng ml^−1^. Using these, we estimated mean sensitivity for the PSA cut-off of 3.0 ng ml^−1^ for each age group, and from these, the MST by age. Results are shown in Table 2. The estimated MSTs were 11.3 and 12.6 years for men aged 50–59 and 60–69 years, respectively.

The probability of overdiagnosis of screen-detected prostate cancer increased with age, from 10% (95% CI 7–11%) for the age group 50–54 to 31% (95% CI 26–32%) for the age group 65–69 (Table 3). The lower estimates of overdiagnosis at earlier ages reflect the greater life expectancy within which the disease could become symptomatic. Overall, 8 out of 1000 men aged 50–69 years undertaking PSA testing are likely to be overdiagnosed.

Table 4 shows the estimates of the proportion of prostate cancer that could be identified at screening, by age group and interscreening interval. For an interscreening interval of 2 years, the proportion of cases detected by screening varied from 86 to 92% for the age groups 50–54 to 65–69, respectively. Increasing the interscreening interval to 10 years, the proportion of cases identified decreased; 55 and 66% for the age groups 50–54 and 65–69, respectively.

The corresponding estimated relative risks of advanced stage prostate cancer are shown in Table 5. For different interscreening intervals, as age increased, the percentage reduction in advanced stage disease decreased. After accounting for overdiagnosis, the percentage reduction in advanced stage disease was smaller. The greatest reductions were estimated for the youngest group, 50–54, ranging from 34% (95% CI 26–42%) for 10-yearly screening starting in this age group to 54% (95% CI 41–65%) for 2-yearly screening. The smallest reductions were seen in the age group 65–69: 6% (95% CI 2–10%) and 9% (95% CI 0–17%) reduction with interscreening intervals of 10 and 2 years, respectively. The reduction in advanced stage disease was more strongly dependent on the interscreening interval for younger age groups, due to the lower test sensitivity in these age groups.

In other way, the findings indicate that potentially, 1.4, 1.8, and 2 advanced stage cancers could be avoided per 1000 men, aged 50–69 years, undertaking PSA testing at 10, 5, and 2 years intervals, respectively. After accounting for overdiagnosis, the potential number of avoided advanced cancer would be reduced to 0.7, 0.9, and 1 per 1000 PSA-tested men, respectively.

## Discussion

There is considerable opportunistic PSA testing activity ongoing in the developed world. Although randomised trial evidence on the effect of such testing on mortality from prostate cancer is not yet available, trials will be reported in the near future. It is therefore appropriate to address implementation issues such as different frequencies of testing and the likely benefits and harms, notably the reductions in advanced disease and the rates of overdiagnosis. For the latter purposes it is necessary to estimate the sojourn time, that is, the potential lead time achieved.

We have demonstrated a simple approach to estimate sojourn time, and overdiagnosis from prevalence screening and population incidence data. Our method of estimation of the MST takes account of the changing underlying incidence with age. Our results suggest that for men aged 50–69 years, the MST of prostate cancer is between 11.3 and 12.6 years. These findings are in line with estimates obtained from studies of prevalence to incidence ratios (Etzioni *et al*, 1998; Auvinen *et al*, 2002), and simulation models (Draisma *et al*, 2003; Pinsky *et al*, 2006). Etzioni *et al* (1998) reported average sojourn time of 11.6 years using prevalence estimates based on autopsy studies and the age-specific incidence of clinical disease from Surveillance Epidemiology, and End Results. Auvinen *et al* (2002) estimated mean lead time and then inferred the MST, assuming that cancers in the first round of screening are detected on average in the midpoint of the MST. The estimates of Auvinen *et al* (2002), based on 292 cases derived from the Finnish screening trial and expected incidence of prostate cancer, were 14.4 years (based on age-adjusted expected incidence) and 9.3 years (based on age-cohort-adjusted expected incidence). Draisma *et al* (2003), using microsimulation modelling based on 151 screen-detected cases derived from the results of the Rotterdam section of the European Randomised study of Screening for Prostate Cancer (ERSPC), showed MST of 12.7 years (range 12.1–14.2 years). However, Draisma *et al* (2003) estimated the MST as the time from screen detection to either clinical diagnosis in the absence of screening or to death by other causes. Pinsky *et al* (2006) estimated MST of 16 years among the participants of the control arm of the Prostate cancer Prevention Trial using convolution model; this higher value could be related to adopted broader definition of clinical cases that included men with positive digital rectal exam findings.

In our study, MST did not vary with age in the age range 50–69 years. The estimates of MST reported by Auvinen *et al* (2002), for the ages at screening between 55 and 67 years, did not vary with age; the age-specific values ranged from 15.8 to 14.6 years and 9.8 to 8.5 years (depending on the reference rates used). On the other hand, Etzioni *et al* (2008) derived simulation-based estimates showing decreasing MST with age, with estimates of 13.7 years (range 12.9–14.5 years) for 50–59 years and 9.1 years (range 8.7–9.6 years) for 60–69 years. This estimate of MST is based on screen-detected cancers that would progress to clinical disease within the lifetime of individuals. This could account for the observed decrease in MST with age. In our study, while estimating MST we did not exclude overdiagnosed cases. Our slightly longer estimates of sojourn time in the 60–69 age group are consistent with the higher prevalence of prostate cancer at older age.

As there will be some asymptomatic PSA testing in the population, our sojourn times and overdiagnosis rates may be underestimated. In a sensitivity analysis, based on the findings of Pashayan *et al* (2006), we estimated the expected number of men in England undertaking PSA testing and the proportion of the prostate cancer diagnosis following PSA testing. Re-estimating MST, after excluding those cases from the incidence data obtained from ONS, increased the sojourn time by only 0.1 year. Any bias in overdiagnosis rates is similarly likely to be small.

Our estimates of MST were derived from prevalence and incidence data, taking into account the sensitivity of the PSA test estimated from external data. Simultaneous derivation of the MST and sensitivity of the PSA test would be preferable. As sensitivity of PSA could not be derived from the ProtecT study, due to absence of data on interval cancers, age-specific sensitivity values were derived from the medical literature. We have estimated an overall PSA test sensitivity of 80%, which is comparable to Hakama *et al* (2007) estimate of 85% using the incidence method and based on randomised prostate cancer screening trial in Finland.

Published PSA sensitivity values were based on sextant biopsy. In the ProtecT study, PSA-detected cases were identified with 10-core biopsy, which is known to have higher detection rate (Stamatiou *et al*, 2007). Thus it is likely that the sensitivity values we have used are underestimates, in which case, the derived MST values are likely to be overestimates.

Our results suggest that overdiagnosis increases with age, partly due to our assumption of a homogeneous model of sojourn time within each age group, and to the fact that the observed prevalence to incidence ratio was similar in all age groups. Because of this, the long sojourn time estimated for all age groups means that the older subjects are more likely to die of other causes before symptomatic diagnosis. In future we plan to model the sojourn time as a mixture of populations within each age group. This may give more substantial overdiagnosis estimates at younger ages.

We estimated that 10–31% of the screen-detected cases would not have been diagnosed in the absence of screening. Here we have defined overdiagnosis as the detection of tumours, which would never have been diagnosed in the absence of screening (Paci *et al*, 2004). There are other measures, such as the number of tumours detected, which did not result in saving of life (McGregor *et al*, 1998). The latter estimate would be a higher proportion of tumours than that estimated using our definition (Frankel *et al*, 2003). This quantity will be the subject of future research on this cohort. Draisma *et al* (2003) reported higher estimates (27–47%) for ages 55–67 years, but apply specifically to the 1991 situation in the Netherlands with respect to clinical detection of prostate cancer. Telesca *et al* (2008), defining probability of overdiagnosis for a screen-detected case as the probability of dying of other causes during the lead time and using simulation modelling, reported 3–14% overdiagnosis. However, these estimates were for a simulated mean lead time of 4.5 years.

Our results indicate that with a 2-year screening interval, 85% of prostate cancer cases could be screen detected. The estimated benefit from screening, in terms of reducing advanced stage disease, ranged from 37% at ages 65–69 years to 67% at ages 50–54 years. When excluding overdiagnosed cases, the estimated benefit was further reduced to 9 and 54% for the age groups 65–69 and 50–54 years, respectively. Etzioni *et al* (2008), in a population-based simulation model, projected a 52% decline in distant stage incidence with PSA screening.

Further research is needed to assess the implications of stage shift on mortality reduction. ERSPC (Draisma *et al*, 2003), Prostate, Lung, Colorectal and Ovarian Cancer Screening Trial (Andriole *et al*, 2005), and the Comparison Arm for ProtecT (Metcalfe *et al*, 2008) in the upcoming years will provide a definite answer on the benefits of prostate cancer screening in reducing mortality.

The figures in Table 5 suggest that screening at later ages (in particular from age 65) may have only a minor effect on incidence of advanced stage disease after taking overdiagnosis into account. They also suggest that the frequency of screening becomes less influential on the incidence of advanced disease with increasing age. This is apparently a result of poorer screening sensitivity rather than shorter sojourn time in the younger age groups. Another target for future research is estimation of the absolute benefit of screening at different ages, and the absolute incremental benefit of screening at earlier ages in addition to later ages. For example, for the men aged 65–69 years at screening in our study, what would have been the additional benefit of a screen 5 years earlier?

Our estimates were based on1544 PSA-detected cases, and on a testing strategy that had a PSA cut-off of 3 ng ml^−1^ and 10-core biopsy. Though there is no screening programme in the United Kingdom, there is *ad hoc* PSA testing (Pashayan *et al*, 2006). Thus, our incidence data also included patients diagnosed by PSA testing, although not in the context of formal screening. Incidence data depend on the frequency of PSA testing, prostate biopsy, and the biopsy protocol. Despite these limitations, our estimates are broadly comparable to published estimates derived from Europe and the United States of America.

In the absence of a screening programme and ongoing *ad hoc* PSA testing in the United Kingdom, our findings give indications to the natural history of prostate cancer and have implications for design of demonstration projects and research studies, pending the results from randomised controlled screening trials. Our results indicate that for men aged 50–69 years, the MST does not vary with age. The proportion of cases that can be detected by screening increases with age. However, the benefit of screening in reducing advanced stage disease is limited by overdiagnosis, which is greater at older ages.

## Figures and Tables

**Table 1:** Number of persons screened, prostate cancers detected by age and stage, including percentages of late stage cases based on the ProtecT study, and the corresponding percentages for clinical prostate cancers from the Eastern Cancer Registry and Information Centre (ECRIC), 2002–2005

	**Age group**				
**Quantity**	**50–54**	**55–59**	**60–64**	**65–69**	**Total**
Number of persons screened^a^	12 092	13 787	10 060	7903	43 842
Total cancers detected^a^	136	347	498	563	1544
Localised stage cancers detected^a^	125	310	430	460	1325
Advanced stage cancers detected^a^	9	35	61	95	200
Stage unknown^a^	2	2	7	8	19
% Advanced stage (preclinical)^a^	6.6	10.1	12.2	16.9	13.0
% Advanced stage (clinical)^b^	26.1	22.5	27.9	28.7	26.9

a Data derived from ProtecT study.

b Data derived from ECRIC.

**Table 2:** Estimates of sensitivity, mean sojourn time, transition rates, and 95% confidence intervals (CI) by age group, based on prevalence screening cancers and registered incident clinical cases, England, 2002–2005

	**Age group**			
	**50–54**	**55–59**	**60–64**	**65–69**
PSA test sensitivity	0.70	0.76	0.83	0.90
Prevalence of preclinical prostate cancer per 10^5^ men	1124.7	2516.9	4950.3	7123.9
Cumulative incidence of prostate cancer per 10^5^ person-years 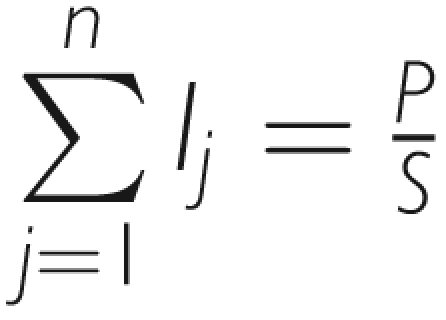	1606.7	3303.0	5981.4	7897.9
Mean sojourn time (years) (95% CI)	11.3 (10.4–12.2)	11.3 (10.8–12.2)	12.6 (11.8–13.4)	12.6 (11.2–13.5)
Transition rate (preclinical to clinical) (95% CI)	0.088 (0.082–0.096)	0.088 (0.082–0.093)	0.079 (0.075–0.085)	0.079 (0.074–0.089)

Abbreviation: PSA=prostate-specific antigen.

**Table 3:** Expected remaining lifetime and probability of overdiagnosis and 95% confidence intervals (CI) by age group

	**Age group**			
	**50–54**	**55–59**	**60–64**	**65–69**
Expected remaining lifetime (years)^a^	27.3	23.0	18.9	15.2
Probability of overdiagnosis (95% CI)	0.10 (0.07–0.11)	0.15 (0.12–0.15)	0.23 (0.20–0.24)	0.31 (0.26–0.32)

a Based on life table on UK males, 2003–2005, produced by the Government Actuary's Department.

**Table 4:** Estimated proportion of prostate cancer to be diagnosed by screening and 95% confidence intervals (CI) by age group for different interscreening intervals

	**Proportion of prostate cancer identified by screening**			
	**Age group**			
	**50–54**	**55–59**	**60–64**	**65–69**
10-year screening interval (95% CI)	0.55 (0.52–0.57)	0.58 (0.56–0.59)	0.63 (0.61–0.64)	0.66 (0.63–0.68)
5-year screening interval (95% CI)	0.71 (0.69–0.73)	0.74 (0.73–0.75)	0.78 (0.77–0.79)	0.80 (0.79–0.82)
2-year screening interval (95% CI)	0.86 (0.85–0.87)	0.88 (0.87–0.88)	0.90 (0.90–0.91)	0.92 (0.91–0.92)

**Table 5:** Predicted relative risk of advanced stage prostate cancer and 95% confidence intervals (CI) by age group and interscreening interval, with and without accounting for overdiagnosis

	**Relative risk of advanced stage**			
	**50–54**	**55–59**	**60–64**	**65–69**
*Assuming no overdiagnosis*				
10-year screening interval (95% CI)	0.60 (0.52–0.70)	0.68 (0.60–0.77)	0.64 (0.58–0.71)	0.73 (0.66–0.81)
5-year screening interval (95% CI)	0.48 (0.38–0.61)	0.59 (0.49–0.70)	0.56 (0.48–0.64)	0.67 (0.59–0.77)
2-year screening interval (95% CI)	0.37 (0.25–0.54)	0.51 (0.40–0.65)	0.49 (0.40–0.59)	0.63 (0.54–0.74)
				
*Accounting for overdiagnosis*				
10-year screening interval (95% CI)	0.66 (0.58–0.74)	0.77 (0.70–0.83)	0.79 (0.75–0.83)	0.94 (0.90–0.98)
5-year screening interval (95% CI)	0.55 (0.46–0.66)	0.70 (0.62–0.79)	0.74 (0.68–0.80)	0.92 (0.87–0.97)
2-year screening interval (95% CI)	0.46 (0.35–0.59)	0.64 (0.55–0.75)	0.70 (0.64–0.76)	0.91 (0.83–1.00)

**Table A1 tbla1:** 

**Paper**	**Age**	**Study size**	**PSA cut-off (ng ml^−1^)**	**Sensitivity %**	**Study setting**
Stenman *et al* (1994)					Retrospective analysis of serum PSA in case–control study (Finland)
	54	5912	3	58	
	71	1292	3	55	
	54	5912	4	58	
	71	1292	4	55	
el-Galley *et al* (1995)					Linking PSA to prostate biopsy data (USA)
	45	168	4	20	
	55	868	4	80	
	65	1929	4	84	
	75	1402	4	87	
					
					
					
Hoffman *et al* (2002)					Community-based, linking PSA to prostate biopsy in ROC analysis (USA)
	55	466	3	89	
	75	825	3	93	
	65	1153	3	89	
	65	1153	3	89	
	75	825	4	90	
	45	176	4	75	
	65	1153	4	84	
Mistry and Cable (2003)					Meta-analysis
Bretton (1994)	65	1027	4	67	Community-based study linking PSA to prostate biopsy data (USA)
Jubelirer *et al* (1994)	68	142	4	100	Community based study with 1-year follow-up (USA)
Imai *et al* (1994)	64	1680	4	73	Mass screening with PSA (Japan)
Imai *et al* (1995)	65	3276	4	80	Mass screening with PSA (Japan)
Higashihara *et al* (1996)	71	701	4	92	Clinical trial – ROC analysis (Japan)
Gustafsson *et al* (1998)	63	1782	4	80	PSA offered to randomly selected men (Sweden)
Brett (1998)	65	211	4	67	General-practice-based linking PSA to prostate biopsy result (Australia)

## Appendix A

### Description of the studis used to derive age-specific sensitivity of PSA test

[Table tbla1]
